# Influence of coronal-morphology of endplate and intervertebral space to cage subsidence and fusion following oblique lumbar interbody fusion

**DOI:** 10.1186/s12891-022-05584-3

**Published:** 2022-07-04

**Authors:** Tianhang Xie, Liming Pu, Long Zhao, Yufei Lu, Zhiqiang Yang, Xiandi Wang, Yueming Song, Jiancheng Zeng

**Affiliations:** 1 Department of Orthopedics, Orthopedic Research Institute, West China Hospital, Sichuan University, 37# Wuhou Guoxue road, Chengdu, 610041 China; 2 Department of Medical Statistics, School of Public Health, Sun Yat-Sen University, Guangzhou, China

**Keywords:** OLIF, Cage subsidence, Fusion, Endplate, Intervertebral space

## Abstract

**Background:**

Endplate morphology is considered to be one of the influencing factors of cage subsidence after lumbar interbody fusion (LIF). Previous radiographic evaluations on the endplate mostly used sagittal X-ray or MRI. However, there are few studies on the CT evaluation of the endplate and intervertebral space (IVS), especially the evaluation of coronal morphology and its influence on subsidence and fusion after LIF. We aimed to measure and classify the shapes of the endplate and IVS using coronal CT imaging and evaluate the radiographic and clinical outcomes of different shapes of the endplate/IVS following oblique lateral lumbar interbody fusion (OLIF).

**Methods:**

A total of 137 patients (average age 59.1 years, including 75 males and 62 females) who underwent L4-5 OLIF combined with anterolateral fixation from June 2018 to June 2020 were included. The endplate concavity depth (ECD) was measured on the preoperative coronal CT image. According to ECD, the endplate was classified as flat (< 2 mm), shallow (2–4 mm), or deep (> 4 mm). The L4-5 IVS was further classified according to endplate type. The disc height (DH), DH changes, subsidence rate, fusion rate, and Oswestry Disability Index (ODI) in different endplate/IVS shapes were evaluated during 1-year follow up.

**Results:**

The ECD of L4 inferior endplate (IEP) was significantly deeper than that of L5 superior endplate (SEP) (4.2 ± 1.1 vs 1.6 ± 0.8, *P* < 0.01). Four types of L4-5 IVS were identified: shallow-shallow (16, 11.7%), shallow-flat (45, 32.9%), deep-shallow (32, 23.4%), and deep-flat (44, 32.1%). A total of 45 (32.9%) cases of cage subsidence were observed. Only one (6.3%) subsidence event occurred in the shallow-shallow group, which was significantly lower than in the other three groups (19 shallow-flat, 6 deep-shallow, and 19 deep-flat) (*P* < 0.05). Meanwhile, the shallow-shallow group had the highest fusion rate (15, 93.8%) and the highest rate of reach minimal clinically important difference (MCID) ODI among the four types. For a single endplate, the shape of L4 IEP is the main influencing factor of the final interbody fusion rate, and the shallow shape L4 IEP facilitates fusion ( OR = 2.85, *p* = 0.03). On the other hand, the flat shape L5 SEP was the main risk factor to cage subsidence (OR = 4.36, *p* < 0.01).

**Conclusion:**

The L4-5 IVS is asymmetrical on coronal CT view and tends to be fornix-above and flat-down. The shallow-shallow IVS has the lowest subsidence rate and best fusion result, which is possibly because it has a relatively good degree in matching either the upper or lower interface of the cage and endplates. These findings provide a basis for the further improvements in the design of OLIF cages.

**Supplementary Information:**

The online version contains supplementary material available at 10.1186/s12891-022-05584-3.

## Introduction

The oblique lateral lumbar interbody fusion (OLIF) technique achieves the effect of increasing disc height (DH) by employing an enlarged cage, thus reaching the goal of decompression of the neural elements by increasing the size of the intervertebral foramen and thinning the posterior ligamentum. This indirect decompression technique was first reported in 2012 [1] and is widely used because it is minimally invasive and effective.

Maintaining the height of the increased intervertebral space and promoting early intervertebral fusion is vital in maintaining the surgical efficacy of OLIF. However, cage subsidence is the most common complication of OLIF, with an incidence of 10.1%-35.3% [2, 3]. In addition, subsidence has a negative effect on interbody fusion [4]. Thus, preventing postoperative cage subsidence and promoting early intervertebral fusion are important in improving the OLIF technology system.

Cage subsidence is an outcome that is comprehensively affected by multiple factors [3, 5–8]. The main influencing factors reported include osteoporosis, intraoperative endplate injury, endplate morphology and DH overdistraction. Countermeasures include strengthening the bone by applying bone cement and protecting the anatomical integrity of the endplate through refined operations. However, with respect to endplate morphology, the interface matching between the endplate and cage may be the fundamental factor that affects the results [9].

At present, the radiological evaluation of lumbar endplates and the intervertebral space (IVS) mostly uses sagittal views on X-ray or MRI to assess the clinical manifestations and the degree of degeneration in lumbar disc disease [10, 11]. However, for evaluating the contact relationship between the endplate and the cage, such as subsidence and bone union, CT images seem to be more suitable. Especially in OLIF, which uses a larger cage with a direction of implantation consistent with the coronal endplate, the CT coronal image seems to be equivalent to or even better than the sagittal plane view. To our knowledge, there are currently few studies using CT images to evaluate the shape of the lumbar endplate and IVS and exploring the correlation between the shape and the cage subsidence and fusion after OLIF. The purpose of the current study was to classify the coronal shape of the lumbar endplate and IVS and evaluate the radiographic and clinical outcomes of different shapes of endplate/IVS to provide a basis for improved design of the OLIF cage in the future.

## Materials and methods

This was a retrospective study that was approved by the institutional review board in our hospital. Patients with L4-5 lumbar disc disease who underwent OLIF by a single chief surgeon from June 2018 to June 2020 were included. The clinical and radiology data were collected and evaluated at preparation and at 1 day, 3 months, 6 months and 12 months after surgery. The inclusion criteria were 1) age from 20 to 80 years with L4-5 degenerative instability or spondylolisthesis; 2) patients without a history of lumbar fracture, bone tumors, cancer metastasis, or previous lumbar surgeries; and 3) without preoperative endplate sclerosis, defect, or Schmorl’s nodes detected on lumbar CT images. The exclusion criteria were as follows: 1) L4-5 degenerative spondylolisthesis ≥ II°, 2) endplate injuries were found intraoperatively or determined on 1 day postoperative CT images, and 3) follow-up period < 12 months. We measured and classified the endplate and L4-5 IVS by preoperative coronal CT imaging and compared the cage subsidence, fusion, and clinical results with the different types. Every patient's radiographs were measured by two independent surgeons. A senior surgeon made an independent judgment when there was any disparity between the two independent surgeons.

### Surgical procedure

OLIF combined with anterolateral screw fixation [12] was used in all patients. Briefly, the left anterolateral side of the L4-5 intervertebral disc was exposed after the three muscular layers were bluntly split. Discectomy and endplate preparation were performed. Then, a polyetheretherketone cage (Clydesdale, Medtronic, USA) of suitable length and height filled with artificial bone (2 g, rhBMP2-loaded calcium phosphate cements, Rebone Co., Ltd. Shanghai, China) was inserted vertically into the intervertebral space. All the cages were 18 mm wide and with a 6° lordotic angle. The length was determined by measuring the left-to-right diameter of the target intervertebral space on preoperative X-ray, which allowed the cage to ride over the epiphyseal ring and without exceeding the lateral edge of the vertebral body. The height was determined by measuring the height of the target intervertebral space on preoperative lateral X-ray and intraoperative sequential trail mold-implant testing. After that, two vertebral screws were inserted and fixed at the lateral side close to the L4 and L5 vertebral bodies, respectively. A connecting rod was placed to lock the screws.

### Radiographic evaluation

**Fig. 1:**
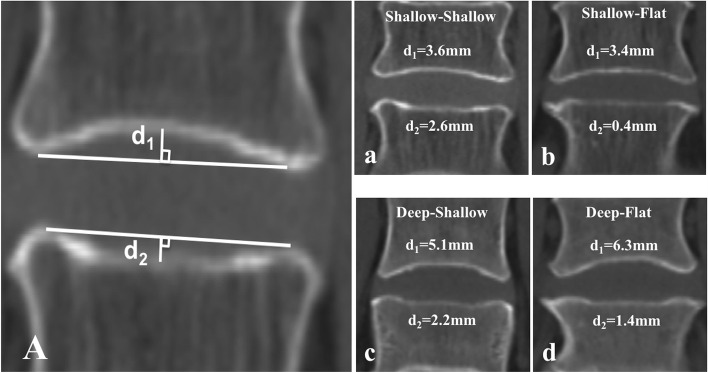
**A**) Endplate concavity depth (ECD) measured by coronal CT imaging (the level corresponds to the mid-sagittal image) from the concavity apex to the line connecting the left and right apex of the endplate. d_1_ and d_2_ represent the ECD of L4 IEP and L5 SEP respectively. (**a**-**d**) The four types of L4-5 intervertebral space (IVS)

The preoperative coronal CT image was selected, and the level corresponding to the mid-sagittal image was confirmed. The endplate concavity depth (ECD) of the L4 inferior endplate (IEP) and L5 superior endplate (SEP) were measured using the distance from the concavity apex to the line connecting the lower/upper left and right apex of the endplate (Fig. 1A). According to the ECD, three types of endplate shapes were defined: flat, ECD < 2 mm; shallow, 2 mm ≤ ECD ≤ 4 mm; and deep, ECD > 4 mm. According to the different endplate shapes, the shape of the L4-5 IVS was classified as X–Y (X indicating the shape of L4 IEP and Y indicating L5 SEP).

**Fig. 2:**
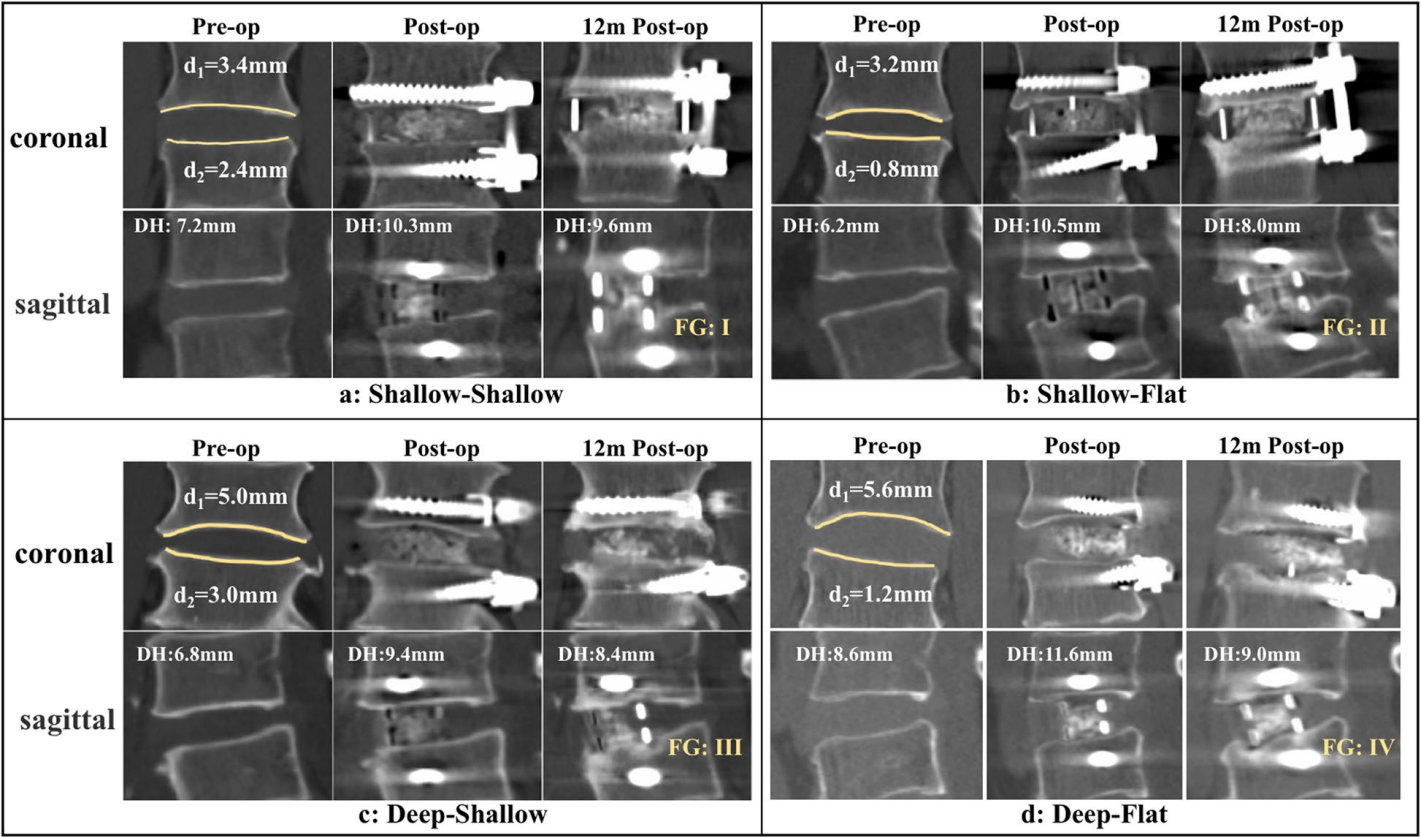
The four typical types of L4-5 IVS and their radiological outcomes. At 12 months postoperatively, the shallow-shallow IVS (**a**) without subsidence and achieving fusion; (**b**) the shallow-flat IVS with subsidence and fusion; (**c**) the deep-shallow IVS without subsidence but not achieving fusion; (**d**) deep-shallow IVS with subsidence and not achieving fusion. DH = disc height; FG = fusion grade

The mid-sagittal CT image was confirmed to evaluate cage position, cage subsidence, and fusion. The cage position was defined as the ratio of the distance from the front marker of the cage to the front edge of the L5 SEP to the length of the L5 SEP [13] (Supplemental Fig. 1). L4-5 disc height (DH) was measured [14] preoperatively and during follow-up. Cage subsidence was defined as more than 2 mm of DH loss after surgery [15]. At 12 months postoperatively, the fusion outcomes were evaluated by the Bridwell classification [16]. Grades I and II were considered fusion, with continuous trabecular bone formation at the upper and lower interface of the endplate and OLIF cage. Grades III and IV were considered nonfusion and lacked continuous trabecular bone at the upper or/and lower interface (Fig. 2).

### Clinical assessment

The Oswestry Disability Index (ODI) was evaluated preoperatively and 3 months, 6 months, and 12 months postoperatively. The minimal clinically important difference (MCID) was used to determine clinical improvement, defined as ODI reduced to 12.8 [17].

### Statistical analysis

Continuous and categorical variables were described as the mean and standard deviation (SD), number and percentage, respectively. We used the Student's t-test and Fisher's exact test to compare the difference in ECD and shapes of the endplate between L4 IEP and L5 SEP. We performed ANOVA and Pearson's chi-square test to compare the difference in the baseline characteristics, fusion rate and Meet MICD ODI among the four shapes of L4-5 IVS. For the repeated measurement variables, such as disc height, changes of DH, cage subsidence and ODI, the generalized estimating equation (GEE) with an autocorrelation matrix was used to determine the effect of time, group and group*time interaction. If the group*time interaction is statistically significant, the simple effect of time and group will be tested. If not, we reported the main effect of time and group. The Bonferroni method was used to adjust the *P*-value for all multiple comparisons. The odds ratio (OR) with 95% confidence interval (95% CI) was calculated to assess the association between the shape of the endplate and subsidence and fusion outcomes. The significance level was set at *P* < 0.05. All statistical analyses were performed using the Statistical Package for the Social Science (SPSS), version 25.0.

### Outcome

A total of 159 patients were admitted, 22 of thom were excluded because they did not meet the inclusion criteria. Finally, a total of 137 patients (75 male/62 female) who underwent L4-5 OLIF were included in the study. The mean age, body mass index (BMI), and bone mineral density (BMD, which was represented by the minimum T score obtained from the hip using dual-energy X-ray absorptiometry scans) were 59.1 ± 12.0 years, 25.2 ± 2.8 kg/cm^2^, and -1.4 ± 0.7, respectively. At 1 day postoperatively, the cage position on mid-sagittal CT imaging was 24.5% ± 7.3% (Table 2).

#### Radiological results

The distribution of the shapes of the endplate and IVS are presented in Tables 1 and 2. The depth of the L4 IEP was significantly deeper than that of the L5 SEP (4.2 ± 1.1 vs 1.6 ± 0.8, *P* < 0.01). Therefore, the composition ratios of shapes are different in the two endplates (*P* < 0.01). The L4 IEP included 44.5% of the shallow shape, 55.5% of the deep, and no flat shape. The L5 SEP included 65.0% flat shape, 35.0% shallow, and no deep shape. Finally, four types of L4-5 IVS shapes (Fig 1 a-d) were described: shallow-shallow (SS) (11.7%), shallow-flat (SF) (32.9%), deep-shallow (DS) (23.4%), and deep-flat (DF) (32.1%). There were no statistically significant differences in baseline characteristics among the patients with the four types of IVS (Table 2).

**Table 1:** The ECD and shapes of L4 IEP and L5 SEP

	L4 IEP	L5 SEP	*p*-value
ECD (mm)	4.2 ± 1.1	1.6 ± 0.8	< 0.01^*^
shapes of endplate (n,%)			
Flat	0 (0.0)	89 (65.0)	
Shallow	61 (44.5)	48 (35.0)	
Deep	76 (55.5)	0 (0.0)	
			< 0.01^#^

*ECD* Endplate concavity depth, *IEP* Inferior endplate, *SEP* Superior endplate

^*^p-value from *t*’test

^#^ *p*-value from pearson chi-square test

**Table 2:** Baseline characteristics of the four shapes of L4-5 IVS

	Total	Shallow-Shallow	Shallow-Flat	Deep-Shallow	Deep-Flat	*p*-value
n (%)	137(100.0)	16 (11.7)	45 (32.9)	32 (23.5)	44 (32.1)	-
Sex (male/female)	75/62	7/9	22/23	18/14	28/16	0.42^*#*^
Age(years)	59.1 ± 12.0	55.2 ± 8.1	59.4 ± 11.9	58.8 ± 14.2	60.4 ± 11.5	0.56*
Body mass index (kg/cm^2^)	25.2 ± 2.8	24.6 ± 3.1	24.9 ± 2.7	24.9 ± 3.3	25.8 ± 2.5	0.35*
Bone mineral density (T score)	-1.4 ± 0.7	-1.4 ± 0.4	-1.5 ± 0.7	-1.3 ± 0.7	-1.4 ± 1.0	0.92*
Cage position(%)	24.5 ± 7.3	26.2 ± 5.8	23.4 ± 8.3	23.7 ± 7.0	25.6 ± 6.9	0.37*

^*^P-value from ANOVA

^#^ *P*-value from pearson chi-square test

**Fig. 3:**
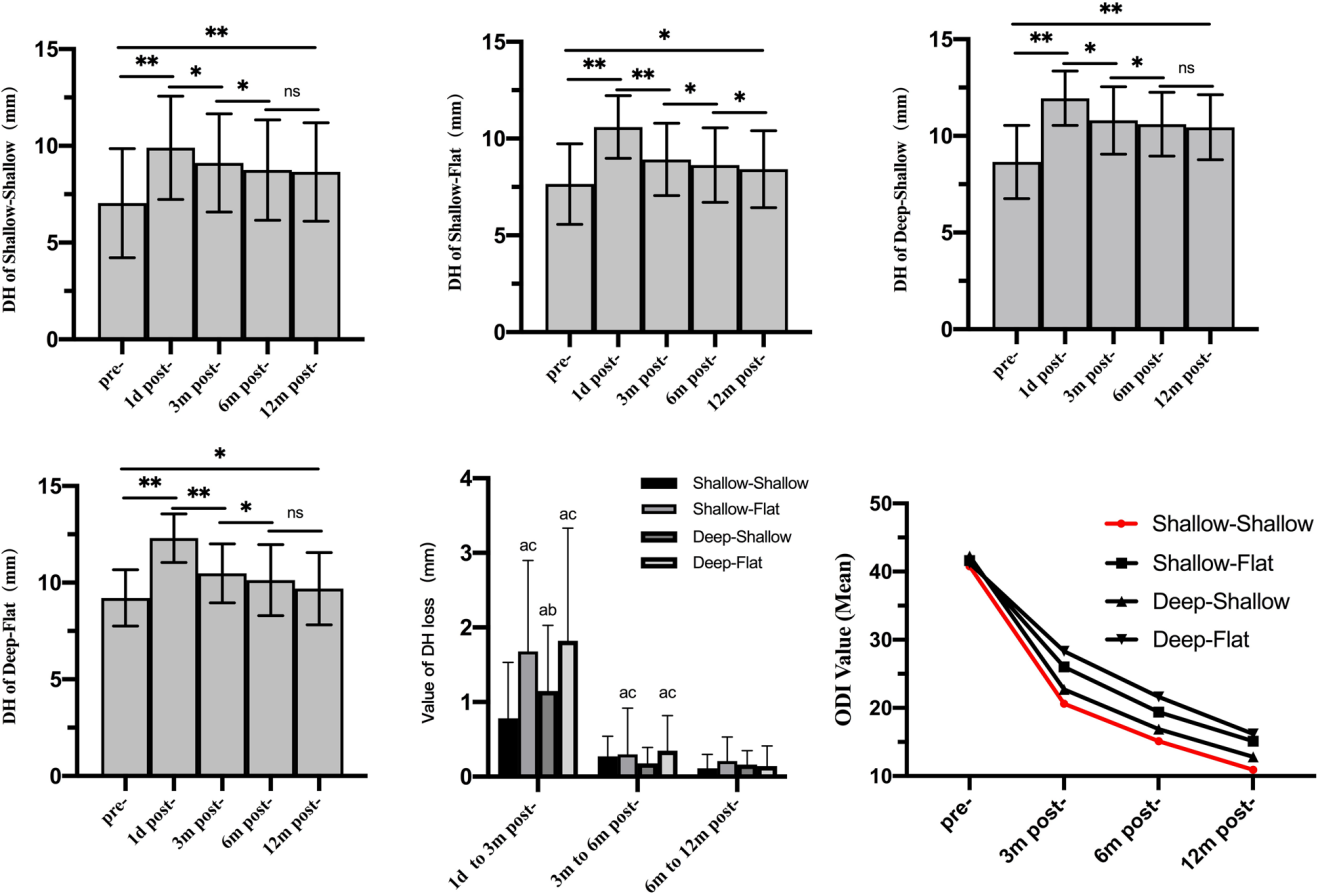
The DH, DH loss and ODI value of four types of L4-5 IVS. * *p* < 0.05, ** *P* < 0.01, a,b,c: *p* < 0.05 for shallow-shallow, shallow-flat, deep-shallow, respectively

A total of 45 (32.9%) instances of cage subsidence were identified (Table 3), of which 42 occurred within 3 months and the remaining 3 cases, two SF and one DF, were identified at 6 months postoperatively. No additional subsidence was identified at 12 months postoperatively. Only 1 case (6.3%) subsidence occurred in the SS group, which was significantly lower than that in the other three groups (*P* < 0.05). According to the data of DH and its changes (Table 3 and Fig. 3), the DH in all groups increased to the same degree(*p* > 0.05)and was significantly higher than preoperatively(*p* < 0.05)at 1 day postoperatively. At 3 months after surgery, the DH showed different degrees of loss in each group. Among them, the SS group lost the least amount, which was significantly less than the other three groups (*p* < 0.05), and the SF and DF groups lost the most and to a similar extent. From 3 to 6 months postoperatively, the degrees of DH loss in the SF and DF groups were still greater than those in the SS and DS groups (*p* < 0.05). Furthermore, all of the lost volume in this interval were smaller than those in the previous interval (1 day to 3 months postoperatively) (*p* < 0.05) within each group. Since then, the overall DH tended to be stable, and only the SF group at 12 months was slightly lower than that at 6 months postoperatively(*p* < 0.05).

**Table 3:** The DH, changes of DH, rate of cage subsidence and fusion of the four shapes of L4-5 IVS

	Total patients (*n* = 137)	Shapes of L4-5 IVS				*p*-value
		Shallow-Shallow	Shallow-Flat	Deep-Shallow	Deep-Flat	
Disc height (DH)(mm)				Pg < 0.01, Pt < 0.01, Pg_*_t < 0.01		
Pre-op	8.3 ± 2.1	7.0 ± 2.8	7.7 ± 2.1	8.7 ± 1.9	9.2 ± 1.5	
1d post-op	11.4 ± 1.9	9.9 ± 2.7*	10.6 ± 1.6*	12.0 ± 1.4*	12.3 ± 1.3*	
3 m post-op	9.9 ± 2.0	9.1 ± 2.5*^#^	8.9 ± 1.9*^#^	10.8 ± 1.7*^#^	10.5 ± 1.5*^#^	
6 m post-op	9.6 ± 2.1	8.9 ± 2.6*^#&^	8.6 ± 1.9*^#&^	10.6 ± 1.7*^#&^	10.0 ± 1.8*^#&^	
12 m post-op	9.4 ± 2.1	8.8 ± 2.6*^#&^	8.4 ± 2.0*^#&@^	10.5 ± 1.7*^#&^	9.9 ± 1.9*^#&^	
Changes of DH(mm)				Pg < 0.01, Pt < 0.01, Pg_*_t < 0.01		
△1 (Postop-preop)	3.1 ± 1.1	2.9 ± 0.6	3.0 ± 1.3	3.3 ± 1.1	3.1 ± 1.1	0.14**
△2 (3 m Postop-postop)	-1.5 ± 1.3	-0.8 ± 0.8	-1.7 ± 1.2^ac^	-1.2 ± 0.9^ab^	-1.8 ± 1.5^ac^	< 0.01**
△3 (6 m Postop-3 m postop)	-0.3 ± 0.5	-0.2 ± 0.3^§^	-0.3 ± 0.6^§ac^	-0.2 ± 0.2^§b^	-0.4 ± 0.5^§ac^	0.03**
△4 (12 m Postop-6 m postop)	-0.2 ± 0.3	-0.1 ± 0.2^§+^	-0.2 ± 0.3^§^	-0.2 ± 0.2^§^	-0.1 ± 0.3^§+^	0.61**
Cage subsidence (n,%)				*Pg* = 0.03, *Pt* = 0.08, Pg_*_t = 0.21		
3 m post-op	42(30.1)	1(6.3)	17(37.8)	6(18.8)	18(41.0)	0.02**
6 m post-op	45(32.9)	1(6.3)	19(42.2)	6(18.8)	19(43.1)	0.00**
12 m post-op	45(32.9)	1(6.3)	19(42.2)	6(18.8)	19(43.1)	0.00**
Fusion Grade (n,%)						
I	18 (13.1)	2 (12.5)	9 (20.0)	3 (9.4)	4 (9.1)	
II	95 (69.3)	13(81.2)	31 (68.9)	23 (71.9)	28 (63.6)	
III	15 (11.0)	1 (6.3)	5 (11.1)	0 (0.0)	9 (20.5)	
IV	9 (6.6)	0 (0.0)	0 (0.0)	6 (18.8)	3 (6.8)	
Fusion rate (n,%)	113(82.5)	15(93.8)	40(88.9)	26(81.3)	32(72.7)	0.13***

*P*_g_, *P*_t_ and *P*_g*t_ represented the the test results of the group main effect, the time main effect and group*time interaction term, respectively,according to the generalized estimating equation with autocorrelation matrix.** the *P*-value represent the testing results for simple effect of with-group at each time point. For the Disc height and change of DH,the simple effect of time was statistically significant(all *P* < 0.001).*** *P*-value from Pearson's chi-square test. All special symbols indicate *P* < 0.05 by Bonferroni correction. Between groups, a: vs shallow-shallow, b: vs shallow-flat, c: vs deep-shallow. Within each group, *: vs preoperative, #: vs 1 day postoperatively, &: vs 3 months postoperatively, @: vs 6 months postoperatively, §: vs △2, + : vs △3

Finally, 113 (82.5%) fusions were identified at 12 months postoperatively, including 18 (13.1%) Grade I and 95 (69.3%) Grade II fusion (Table 3). The compositions of fusion were 15 (94.0%) in the SS group, 40 (88.9%) in the SF group, 26 (81.3%) in the DS group, and 32 (72.7%) in the DF group. The four types of typical cases are shown in Fig. 2.

The analysis of subsidence and fusion outcomes based on single endplate shape is shown in Table 4. The shape of L4 IEP is the main influencing factor of the final interbody fusion rate, in which the shallow-shape L4 IEPs facilitate fusion ( OR = 2.85, 95% CI: 1.1,7.7, *p* = 0.03). On the other hand, the flat- shape L5 SEP was the main risk factor for cage subsidence (OR = 4.36, 95% CI: 1.8,10.8, *p* < 0.01).

**Table 4:** Finally radiological results basis on single endplate

	L4 IEP		*OR* (95%*CI*)	*P*	L5 SEP		OR (95%CI)	*P*
	Shallow(61)	Deep(76)*			Flat(89)	Shallow(48)*		
Cage subsidence (n,%)	20 (32.8)	25 (32.9)	1.00 (0.5,2.0)	0.99	38 (42.7)	7 (14.6)	4.36 (1.8, 10.8)	< 0.01
Fusion (n,%)	55(90.2)	58(76.3)	2.85 (1.1,7.7)	0.03	72 (80.9)	41 (85.4)	0.72 (0.3,1.9)	0.51

^*^Compare group, *P*-value from pearson chi-square test

#### Clinical results

The ODI values are shown in Fig. 3 and Table 5. Patients in each group showed a trend of gradual improvement after surgery (*P* < 0.05). However, there were some differences between groups. The SS group showed the best improvement in ODI at 3 months and 6 months postoperatively (*P* < 0.05). Finally, the values in the SS and DS groups (*P* > 0.05) were less than (*P* < 0.05) those in the SF and DF groups. This trend was similar to that in DH and its loss. At 12 months postoperatively, 93 (67.9%) cases met the MICD criteria, including 15 (93.8) in the SS group, 25 (55.6%) in the SF group, 25 (78.1%) in the DS group, and 28 (63.6%) in the DF group.

**Table 5:** The clinical result (ODI) of the four shapes of L4-5 IVS

	Total patients	Shapes of L4-5 IVS				*p*-value
		Shallow-Shallow	Shallow-Flat	Deep-Shallow	Deep-Flat	
ODI(Mean ± SD)				*P_group* = 0.01; *P_tim*e < 0.01; P_group*time: < 0.01		
Pre-op	41.3 ± 8.4	40.8 ± 9.7	41.6 ± 8.1	42.3 ± 8.5	41.2 ± 8.6	0.99**
3 m Post-op	25.3 ± 7.9	20.6 ± 9.5^*^	26.0 ± 6.8^*ac^	22.7 ± 7.0^*ab^	28.3 ± 7.8^*ac^	< 0.01**
6 m post-op	19.0 ± 6.2	15.1 ± 4.4^*#^	19.4 ± 5.2^*#a^	16.9 ± 5.9^*#b^	21.6 ± 6.7^*#ac^	< 0.01**
12 m post-op	14.4 ± 5.9	10.9 ± 3.2^*#&^	15.1 ± 5.7^*#&ac^	12.8 ± 4.4^*#&b^	16.2 ± 7.1^*#&ac^	< 0.01**
Meet MICD ODI (n,%)	93(67.9)	15(93.8)	25(55.6)	25(78.1)	28(63.6)	0.02***

*P*_g_, *P*_t_ and *P*_g*t_ represented the the test results of the group main effect, the time main effect and group*time interaction term, respectively,according to the generalized estimating equation with autocorrelation matrix..** the *P*-value represent the testing results for simple effect of with-group at each time point. *** *P*-value from Pearson's chi-square test. All special symbols indicate *P* < 0.05 by Bonferroni correction. between groups,*: vs preoperative, #: vs 3 months postoperatively, &: vs 6 months postoperatively. Within each group, a: vs shallow-shallow, b: vs shallow-flat, c:vs deep-shallow

## Discussion

Compared with spinal direct decompression, the indirect decompression based on the principle of increasing the height of the intervertebral space is more necessary to prevent cage subsidence and promote early fusion. Oh [18] believes that cage subsidence following posterior lumbar interbody fusion will not have a clear adverse effect on clinical symptoms, even if subsidence is severe. This may be related to surgical principles. In traditional posterior or transforaminal approaches, the spinal canal can be directly enlarged by removing the ligamentum flavum and part of the bone tissue to achieve nerve decompression. The main functions of the intervertebral cage are to fill the defect caused by the removal of the disc and provide a bridge for the growth of new bone. Even if the height of the intervertebral space is partially lost, the volume of the posterior spinal canal may not be drastically affected. In contrast, for indirect decompression methods such as OLIF and lateral lumbar interbody fusion (LLIF), the expansion of the spinal canal is the concomitant effect of the increase in the height of the intervertebral space. Therefore, in addition to the effect of filling and bridging, the OLIF cage is also critical in maintaining DH, which is one of the most important reasons why the cage in the indirect decompression procedure is designed to be higher and larger than that in direct decompression surgery. Cage subsidence following OLIF has been associated with axial pain and recurrent neurological symptoms [19]. Marchi L [20] further pointed out that a higher degree of subsidence will lead to the worse clinical improvement. Similar results were obtained in the present study. Patients in the SS group with a lower degree of postoperative DH loss had better clinical improvement than other groups with a higher degree of DH loss. This again confirms the mechanism of indirect decompression and the importance of maintaining the height of the intervertebral space after OLIF.

Intervertebral prostheses have undergone decades of development. Its goal has always been to be as close as possible to the normal intervertebral structure in material and shape. In terms of shape, the lumbar intervertebral space is an irregular three-dimensional structure surrounded by upper and lower endplates and involves many parameters, such as length, width, height, depth, and angle [21].. For the complex and diverse shapes, the 3D printing technique is considered the best solution at present. Serra [22] successfully fabricated a lumbar cage using composite materials (POSS-PCU) by 3D printing and obtained suitable mechanical properties similar to those of trabecular bone and with good biocompatibility. Mobbs [23] designed and manufactured through 3D printing an acrylonitrile butadiene styrene (ABS) prosthesis based on real anatomical parameters, which was successfully applied in a patient with L5-S1 disease. These studies have undoubtedly greatly encouraged the medical applications of 3D printing for cages. However, higher costs and stricter requirements for materials and technology may make it difficult to popularize the personalized cages. Therefore, a mass-produced anatomical intervertebral cage may be more promising. At present, the shape of the existing OLIF cage has almost met the anatomical parameters on multiple dimensions such as length, width, height, and lordosis angle. However, on the coronal view, it is designed as a curved surface with the same upper and lower curvature, which is obviously not consistent with the normal intervertebral structure. We find that on coronal CT image, the L4 IEP tends to arc with a deeper depth and the L5 SEP tends to be flat. Even for shallow-shallow IVS, the depths of the L4 IEP and L5 SEP are not exactly the same. This indicates that in the coronal view, the shape of the OLIF cage is different from that of L4-5 IVS, which will inevitably lead to the incomplete matching of the cage and endplate.

**Fig. 4:**
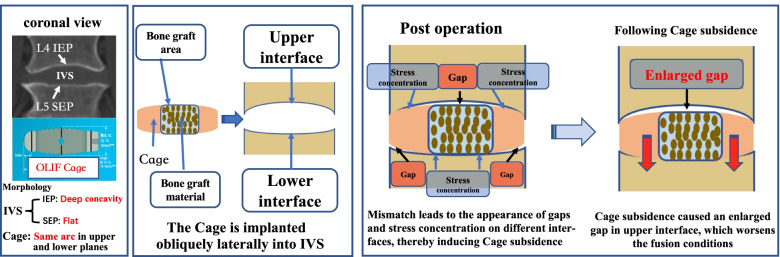
Hypothesis of interface mismatch of the OLIF cage and interverbal space leading to cage subsidence and nonunion

Regardless of two-dimensional or three-dimensional vision, putting two different shapes together will inevitably lead to overlapping and nonoverlapping areas. Based on the morphological difference between the intervertebral space and OLIF cage, the upper and lower interfaces of IVS-cage may overlap differently. That is, the center area of the lower interface may overlap, and the surrounding area may not; in the upper interface, the opposite is true, and a gap may even appear in the center (Fig. 4). Higher local stress is the direct cause of cage subsidence [9, 24]. In terms of the lower interface, poor matching results in increased local stress in the central area. This combined with the effect of gravity may be the main reason why subsidence mainly occurs in the superior endplate [25]. We also found that when the L5 SEP was flat, which means that the lower interface had poor matching, the final cage subsidence rate was significantly higher than when the L5 SEP was shallow. In addition, stress is also important for fusion. Bone formation and remodeling require appropriate stress stimulation. Less stress may cause bone resorption and nonunion [26]. The results indicated that when L4 IEP was the deep type, the fusion rate was lower than if L4 IEP were shallow. The reason may be due to mismatching in the central area of the upper interface, leading to poor stress that does not promote osteogenesis. Furthermore, the extent of bone defect repair cannot be ignored [27]. Subsidence may lead to further deterioration of the upper interface matching and increase the fusion distance, thereby further worsening the conditions of fusion. This may be the important reason for the worst fusion rate in the deep-flat IVS. Consequently, based on the research results, we propose that the interface matching degree of the endplate and cage affects the subsidence and fusion. The lowest subsidence rate and best fusion results are obtained in that shallow-shallow IVS, possibly because it has a relatively good matching degree in either the upper or lower interface. The verification of this hypothesis requires further research.

### Limitations

This study has some limitations. First, this was a retrospective study with a relatively limited cohort and no sample size or power analysis was performed. It might be insufficient to detect significant differences between subgroups in factors such as fusion rate. Therefore, these results need to be validated in a large-scale cohort. Second, the follow-up period was relatively short. Although cage subsidence mainly occurs in the early postoperative period, detecting further changes in DH and fusion requires longer observation. Third, to cage morphology, we unified the width and angle and used the same standard to determine the height and length based on the patients' preoperative imaging data. However, this study did not analyze the effect of cage parameters on the results. Fourth, the surgical technique in this study involved OLIF with anterolateral fixation, so it is possible that result cannot be generalized to OLIF with posterior fixation or standalone OLIF. Finally, endplate-cage matching is a three-dimensional matching relationship. We analyzed only a single plane on CT coronal imaging, which is meaningful but not comprehensive enough. Therefore, prospective multidimensional radiological and clinical observational studies with larger samples and longer follow-up times are needed.

## Conclusion

The L4-5 IVS is the asymmetrical shape on coronal CT view that tends to be fornix-above and flat-down. Different shapes of endplates and intervertebral spaces have different radiological and clinical outcomes after OLIF. The shallow-shallow IVS has the lowest subsidence rate and best fusion result, possibly because it has a relatively good matching degree either in the upper or lower interface of the cage and endplates. This provides a basis for further improvements in the design of the OLIF cage.

## Supplementary Information

**Additional file 1.**

## Data Availability

The analyzed data sets generated during the current study are available from the corresponding author on reasonable request.

## Abbreviations

OLIF: Oblique lateral lumbar interbody fusion
DH: Disc height
IVS: Intervertebral space
ECD: Endplate concavity depth
IEP: Inferior endplate
SEP: Superior endplate
ODI: Oswestry Disability Index
MCID: Minimal clinically important difference
OR: Odds ratio
BMI: Body mass index
BMD: Bone mineral density
SS: Shallow-shallow
SF: Shallow-flat
DS: Deep-shallow
DF: Deep-flat
LLIF: Lateral lumbar interbody fusion
