# Supplementary material for: Influence of coronal-morphology of endplate and intervertebral space to cage subsidence and fusion following oblique lumbar interbody fusion
Source: BMC Musculoskelet Disord. 2022 Jul 4;23:633. doi: 10.1186/s12891-022-05584-3 (PMC9252057; doi:10.1186/s12891-022-05584-3)
Supplement: Supplementary file 1 — Additional file 1. [file 12891_2022_5584_MOESM1_ESM.docx]

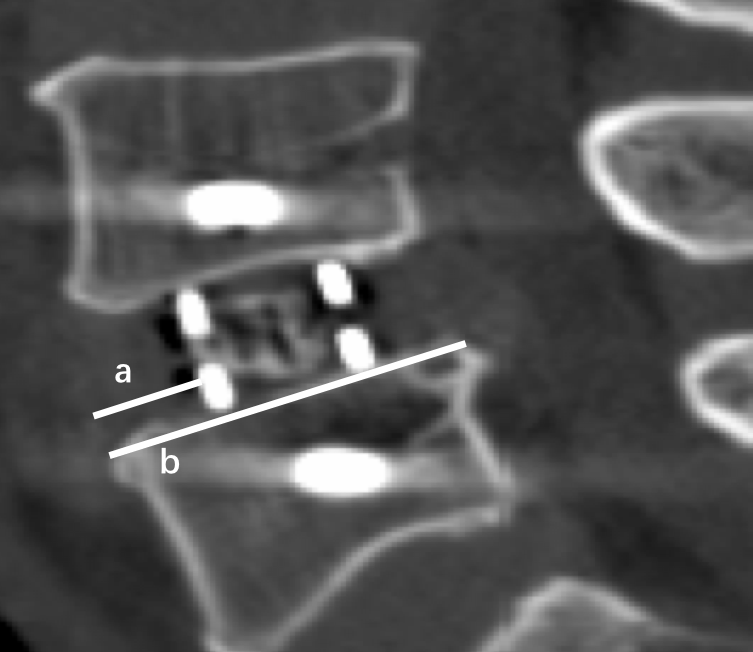


Supplement figure 1.The measurement of cage position. The cage position is calculated as a/b*100%. a, the distance between the anterior metal marker and the leading edge of the caudal endplate. b, the length of caudal endplate
